# Harnessing naturally occurring mutations for T-cell therapy: a potential new avenue to enhance treatment efficacy

**DOI:** 10.1038/s41392-024-01835-y

**Published:** 2024-04-30

**Authors:** Michael Hiltensperger, Jürgen Ruland, Kilian Schober

**Affiliations:** 1 Mikrobiologisches Institut-Klinische Mikrobiologie, Immunologie und Hygiene, Friedrich-Alexander-Universität Erlangen-Nürnberg, Erlangen, Germany; 2 Institute of Clinical Chemistry and Pathobiochemistry, School of Medicine, Technical University of Munich, Munich, Germany; 3 TranslaTUM, Center for Translational Cancer Research, Technical University of Munich, Munich, Germany; 4 German Cancer Consortium (DKTK), Heidelberg, Germany; 5 German Center for Infection Research (DZIF), partner site Munich, Munich, Germany; 6 FAU Profile Center Immunomedicine, Friedrich-Alexander-Universität Erlangen-Nürnberg, Erlangen, Germany

**Keywords:** Tumour immunology, Cancer therapy, Immunotherapy

In a recent study published in *Nature*, Garcia et al. use a sophisticated approach to identify fitness-enhancing mutations for T cells that was inspired by cancer evolution.^1^ The identified *CARD11-PIK3R3* gene fusion enhanced tumor rejection and persistence of engineered T cells in multiple tumor models and might have the potential to improve efficacy of adoptive T-cell therapies in cancer patients.

**Fig. 1:**
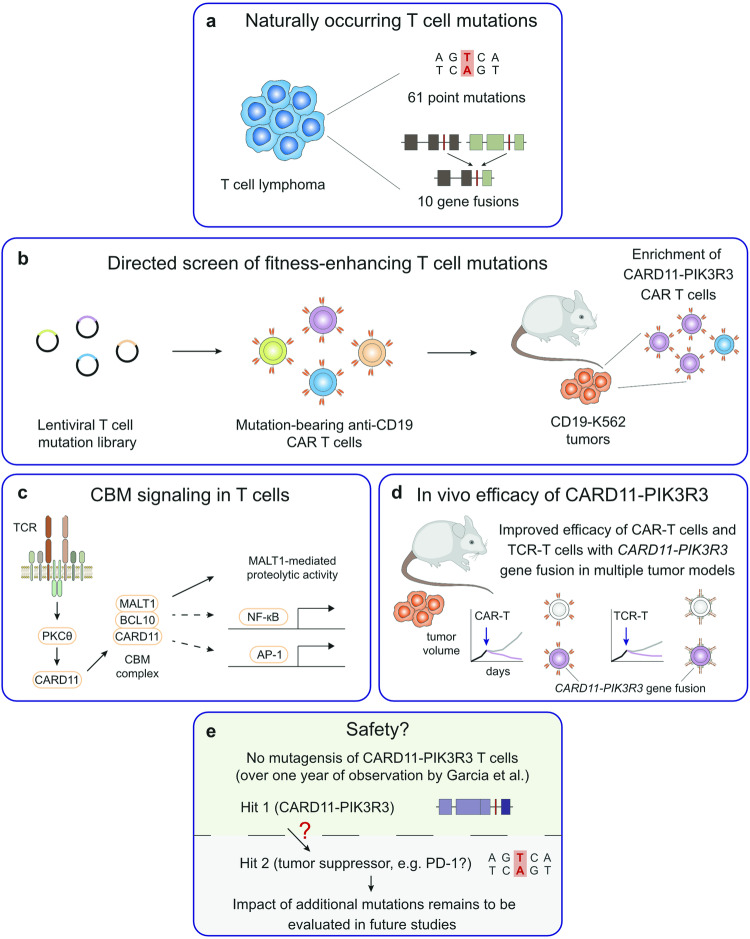
Gene fusion of CARD11-PIK3R3 enhances T-cell tumor rejection in multiple preclinical tumor models. **a** Selection of naturally occurring point mutations and gene fusions from T cell lymphomas. **b** Schematic of part of the screen by Garcia et al. to identify T cell mutations that enhance fitness and persistence of engineered T cells. **c** Schematic of CARD11-BCL10-MALT1 (CBM) complex signaling. **d** Schematic of efficacy testing for *CARD11-PIK3R3* genetic perturbation in engineered CAR T cells and TCR T cells in multiple tumor models. **e** What is known about the safety of *CARD11-PIK3R3* gene fusion in engineered T cells from Garcia et al. (green) and open questions of its impact on mutagenesis in the context of additional mutations like PD-1 (grey)

In an initial screen, the authors selected 61 point mutations and 10 gene fusions from T-cell lymphomas (Fig. 1a) and screened their impact on T-cell signaling and in vivo anti-tumor efficacy in various tumor models. Thereby, the authors identified a gene fusion of *CARD11-PIK3R3* with fitness-enhancing capabilities that was originally discovered in a patient with a CD4^+^ cutaneous T cell lymphoma. Notably, this chimeric gene fusion that contains sequences from the CARD11 scaffold protein and the phosphoinositide-3-kinase regulatory subunit 3 would not have been identified in conventional screens that cover mainly point mutations, highlighting the power of approaches that are based on naturally occurring mutations.

For their initial screen, Garcia et al. used anti-CD19 Chimeric Antigen Receptor (CAR) T cells with a CD28 or 4-1BB costimulatory domain fused to a CD3ζ signaling domain (CD28z or BBz) that were transduced with vectors expressing a set of naturally occurring T cell lymphoma genes and investigated their effects on the activation of NFAT, NF-κB, and AP-1 signaling, IL-2 secretion, and PD-1 expression in vitro upon coculture with CD19-expressing K562 cells. Although a handful of somatic alterations elicited increased reporter signaling in CAR^+^ mutation-bearing Jurkat reporter cells in vitro, enhanced IL-2 secretion was predominantly observed for *CARD11-PIK3R3* gene fusion and *CARD11* point mutations. Subsequently, the authors adoptively transferred human anti-CD19-BBz CAR T cells into mice harboring CD19-expressing K562 tumors and evaluated the persistence of CAR T cells engineered with a specific set of mutations (Fig. 1b). Among the 71 somatic alterations, only five were enriched in the tumors and out of these, *CARD11-PIK3R3* gene fusion was the only genetic perturbation that showed increased NF-κB and AP-1 reporter signaling and IL-2 secretion in vitro. Due to these promising characteristics of the CARD11-PIK3R3 expressing CAR T cells, the authors then studied the signaling pathways that are engaged by the CARD11-PIK3R3 fusion protein in more detail.

The wild-type CARD11 protein is part of the tripartite CARD11-BCL10-MALT1 (CBM) signalosome^2^ that is physiologically assembled in response to T cell receptor (TCR) triggering (Fig. 1c). Subsequently, the CBM complex induces downstream signaling via the NF-κB and AP-1 signaling pathways for T cell activation and, in addition, MALT1 proteolytic activity. The physiological functions of PIK3R3 in T cells are still ill-defined. The CARD11-PIK3R3 fusion protein autonomously enhances CBM complex signaling and the authors have demonstrated that the SH2 domain of PIK3R3 is critical for this process by binding phospho-tyrosine residues. These findings indicate that increased CBM complex signaling in response to antigen-mediated T cell activation could give T cells with *CARD11-PIK3R3* gene fusion an advantage in the tumor microenvironment in vivo.

A significant strength of the study is the thorough investigation of the effects of the *CARD11-PIK3R3* gene fusion in CAR T cells and TCR-specific T cells in multiple tumor models (Fig. 1d). In a xenograft model of acute lymphoblastic leukemia, CARD11-PIK3R3 expressing anti-CD19 CAR T cells showed superior tumor rejection compared to CAR T cells without the gene fusion, even at low input cell numbers. Furthermore, rechallenge of surviving animals with Nalm6 cells without administration of additional anti-CD19-CD28z CAR T cells showed improved efficacy for CARD11-PIK3R3 CAR T cells as well, which could be due to their increased persistence compared to conventional CAR T cells. This hypothesis is supported by the accumulation of CARD11-PIK3R3 anti-CD19-BBz CAR T cells in hCD19-B16 tumors. In addition, the authors also investigated the function of the *CARD11-PIK3R3* gene fusion in anti-MCAM-CD28z CAR T cells that target an additional antigen in M28 tumor-bearing mice. Again, in this model, the authors demonstrated similar improvements in regard to tumor rejection for CAR T cells harboring the *CARD11-PIK3R3* gene fusion. In a comparative setting with ovalbumin (OVA)-specific TCRs, OT-I T cells with *CARD11-PIK3R3* gene fusion outgrew regular OT-I T cells by over 100-fold when co-administered into B16-OVA tumor-bearing mice. Most notably, administration of only 20,000 CARD11-PIK3R3 expressing OT-I cells was sufficient for complete tumor clearance up to 30 days and a complete response in 3 out of 5 animals, while 2 million regular OT-I T cells failed to control tumor growth in the control animals. In addition, in a further rechallenge experiment with B16-OVA melanoma cells, the mice that originally received CARD11-PIK3R3 OT-I T cells could still control tumor growth. Finally, Garcia et al. also investigated another TCR-specificity against the G12D Kras mutation in a xenograft tumor model for gastric cancer. Again, the *CARD11-PIK3R3* gene fusion enhanced the anti-tumor efficacy of Kras^G12D^-specific T cells in comparison to control T cells.

Despite these promising results, utilizing mutations from lymphomas that are potentially oncogenic, triggers some safety concerns. The authors already addressed these concerns to some extent and did not see any signs of malignant transformation of T cells harboring the *CARD11-PIK3R3* gene fusion in their experiments. On top, they performed long-term control experiments with CARD11-PIK3R3 OT-I T cells and evaluated the potential expansion of these cells in different organs after 248 days and in blood samples for up to 426 days (Fig. 1e, top). Again, no signs of malignant transformation or abnormal cell growth of the transferred cells were observed, indicating that *CARD11-PIK3R3* gene fusion does not cause malignant transformation on its own. However, it cannot be excluded that *CARD11-PIK3R3* gene fusion represents a tumor predisposing genetic hit, that could provide the ground for a second or third mutation to induce malignant transformation (Fig. 1e, bottom). This paradigm has been previously shown for the T cell lymphoma fusion kinase ITK-SYK that is, in principle, oncogenic, but in otherwise non-mutated T cells is kept in check by PD-1. However, a genetic loss of *PDCD1*, which encodes PD-1, enables a strong expansion of ITK-SYK^+^ cells and malignant transformation.^3^ The fact that *PDCD1* is often deleted as a tumor suppressor in human T cell lymphomas on the one hand,^3^ and the clinical relevance of PD-1 as a target for immune checkpoint inhibition or genetic ablation in engineered T cells on the other hand, exemplifies how delicate the T-cell intrinsic balance between oncogenic and tumor suppressor signaling is. Therefore, a thorough investigation of each genetic alteration for immunotherapy and the potential risks of additional mutations will be important to be assessed in future studies. In addition, enhanced cytokine production in fitness-enhanced mutated T cells could potentially exacerbate certain adverse events like cytokine release syndrome (CRS) or “on-target, off-tumor cytotoxicity”, which also needs to be addressed in future clinical studies with these genetic mutations.

Garcia et al. also hypothesize that the phenotypic effects of CARD11-PIK3R3 are antigen-dependent and therefore do not lead to autoproliferation in the absence of an antigenic trigger. The fact that enhanced proliferation of *CARD11-PIK3R3* harboring T cells only occurs in response to CAR/TCR signaling is an important safety advantage over some other reported genetic perturbations, such as genetic disruption of the gene that encodes the epigenetic regulator ten–eleven translocation 2 (*TET2*). Although loss of *TET2* led to increased persistence of CAR T cells and complete remission in a patient with chronic lymphocytic leukemia, clonal expansion of *TET2*-deficient CAR T cells is antigen independent and the cells are prone to obtain additional somatic mutations.^4^ Therefore, future studies should explore whether *CARD11-PIK3R3* gene fusion would be a safer approach compared to *TET2*-editing.

A recent evaluation by the FDA of 8000 patients treated with CAR T cells showed around 20 cases of T cell lymphomas.^5^ To put these numbers into perspective, compared to cases with T-cell lymphomas in checkpoint inhibition therapy, the number of cases in patients treated with CAR T cells are overall lower, which suggests a low risk of secondary T cell lymphomas for CAR T-cell therapies in general.^5^ Moreover, the authors propose additional safety strategies that could reduce the risk of malignant transformation or adverse events for *CARD11-PIK3R3* gene fusion and other naturally occurring mutations from lymphomas: (I) knock-out of endogenous TCRs to prevent TCR-mediated immunopathology, (II) inducible expression upon encounter of another tissue-specific antigen in the form of synNotch receptors, (III) directed integration to avoid random integration into tumor suppressor genes, or (IV) the implementation of suicide switches could improve the safety profile of the mutated T cells. Furthermore, the authors indicate that fitness-enhancing mutations like *CARD11-PIK3R3* gene fusion could also circumvent the need for lymphodepletion, which is commonly administered prior to T-cell therapy to enhance the persistence of the transferred T cells, since CARD11-PIK3R3 showed impressive enrichment over unmutated T cells in the absence of lymphodepletion.

Together, Garcia et al. demonstrate with their exciting study that incorporation of fitness-enhancing naturally occurring T-cell mutations could represent an interesting new avenue to enhance T-cell therapies. However, translation into the clinic requires further safety investigations.
